# Horizontal Gene Transfer and Genome Rearrangements Shape Bacterial Adaptation for Bioremediation

**DOI:** 10.1111/1462-2920.70374

**Published:** 2026-07-08

**Authors:** Zaki Saati‐Santamaría, Amando Flores, Inés Canosa, Paula García‐Fraile

**Affiliations:** ^1^ Institute for Agribiotechnology Research (CIALE) Salamanca Spain; ^2^ Unidad de Excelencia Producción, Agrícola y Medioambiente (AGRIENVIRONMENT) Universidad de Salamanca Salamanca Spain; ^3^ Departamento de Microbiología y Genética Universidad de Salamanca Salamanca Spain; ^4^ Laboratory of Fungal Genetics and Metabolism Institute of Microbiology of the Czech Academy of Sciences Prague Czech Republic; ^5^ Universidad Pablo de Olavide Centro Andaluz de Biología del Desarrollo/Consejo Superior de Investigaciones Científicas/Junta de Andalucía Seville Spain; ^6^ Associated Research Unit of Plant‐Microorganism Interaction, USAL‐CSIC (IRNASA) Salamanca Spain

**Keywords:** bacterial evolution, bioremediation, evolutionary ecology, horizontal gene transfer, metabolic HGT hubs, pollutants, xenobiotics

## Abstract

Bacterial bioremediation involves bacterial strains and communities and their complex interactions with the environment, aiming to restore ecological balance by degrading contaminants in natural systems (soil, water, air). These processes rely on coordinated gene clusters encoding catabolic pathways. Many xenobiotic‐catabolic gene clusters (XGCs) reside on mobile genetic elements (MGE), enabling horizontal gene transfer (HGT) and genome rearrangements that drive rapid microbial adaptation to anthropogenic contaminants. Here we review the evolutionary and ecological roles of HGT and genome restructuring in assembling and optimising biodegradative functions. We introduce the concept of *metabolic HGT hubs*—microbial taxa, mobile elements, and ecological features that serve as central nodes for gene exchange—facilitating metabolic innovation and cooperation within microbial consortia. These processes enhance ecosystem resilience and pollutant degradation efficiency by promoting functional redundancy and metabolic division of labour. Understanding these dynamics informs strategies for engineering microbial communities and genetic bioaugmentation to improve bioremediation outcomes. Our perspective highlights bioremediation as an extension of metabolic network evolution under anthropogenic selection, emphasising both its potential and the need to consider ecological and biosafety implications.

## Graphical abstract

Exposure to xenobiotics triggers genome evolution and horizontal gene transfer (HGT), driving the acquisition of novel catabolic functions. These genomic adaptations provide a competitive fitness advantage that reshapes microbial community structure and enhances biodegradation impact.

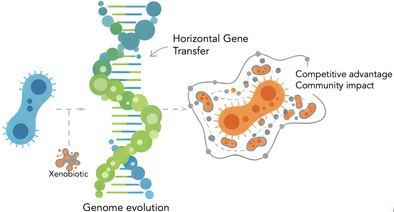

## 1 Introduction

Bioremediation is the degradation, transformation, or detoxification of environmental contaminants through biological systems. Its main mechanisms include redox reactions, biosorption, bioaccumulation, and enzymatic transformations that reduce the toxicity and persistence of hazardous compounds (Azubuike et al. 2016; Filote et al. 2021; Sanjana et al. 2024). Bacterial bioremediation can target a broad range of pollutants, including petroleum hydrocarbons, plastics, dyes, pesticides, pharmaceuticals, and heavy metals such as lead, chromium, cadmium, and arsenic (Bala et al. 2022; Maglione et al. 2024). Although numerous bacterial taxa have demonstrated remarkable tolerance and removal efficiency in soil, water, and wastewater, contaminant bioavailability, which is influenced by the hydrophobicity of many pollutants, remains a major limiting factor. Hydrophobic compounds, such as petroleum hydrocarbons and plastics, are often poorly soluble in water, reducing their accessibility to microbial degradation. Various strategies, such as surfactant supplementation, bioaugmentation, selective biostimulation, and bacterial chemotaxis, have been developed to enhance the accessibility and biodegradation of these hydrophobic compounds (Famisan and Brusseau 2003; Ortega‐Calvo et al. 2013; Posada‐Baquero et al. 2024; Muter 2023).

At the genetic level, catabolic genes responsible for xenobiotic degradation are often organised into *xenobiotic‐catabolic gene clusters* (XGCs) containing enzymes, regulators, and transporters. This genomic arrangement facilitates coordinated transcription, often as operons, ensuring the efficiency of the catabolic pathway. Many XGCs are located on mobile genetic elements (MGEs) such as plasmids, integrative conjugative elements, or transposons, which enable their horizontal transfer among microorganisms and promote rapid adaptation to emerging contaminants (Ríos‐Miguel et al. 2020; Castro‐Gutierrez et al. 2022; Holert et al. 2024).

**FIGURE 1:**
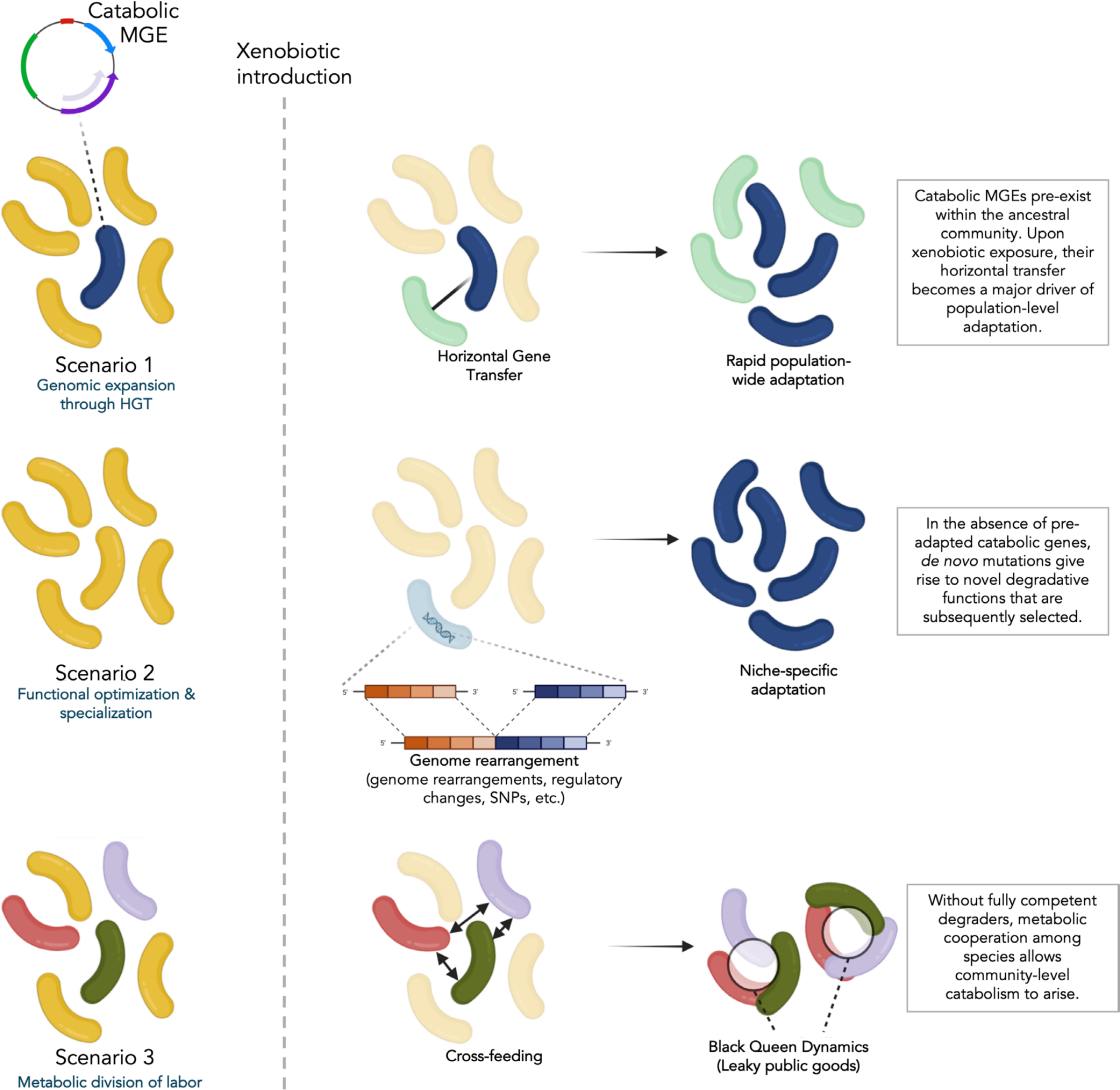
Evolutionary scenarios enabling microbial adaptation to xenobiotic degradation. Scenario 1—Horizontal acquisition of catabolic functions. Within a diverse bacterial population, only a minority carry mobile genetic elements (MGEs) encoding catabolic pathways. Upon xenobiotic exposure, cells lacking these functions experience a fitness collapse, whereas those harboring catabolic MGEs, and those that acquire them via horizontal gene transfer, rapidly dominate the population. Scenario 2—De novo innovation through mutation. In the absence of pre‐existing catabolic genes, spontaneous mutations can generate novel metabolic capabilities. Exposure to the xenobiotic selects for these rare innovators, which subsequently rise to fixation. Scenario 3—Community‐level degradation via metabolic cooperation. When no individual species can fully degrade the xenobiotic, microbial consortia can assemble cooperative networks in which partial degraders engage in cross‐feeding. These emergent functional guilds gain fitness under contamination, enabling community‐level biodegradation. Figure created with BioRender.com.

Bioremediation ultimately relies on microbial communities capable of degrading pollutants through metabolic cooperation and genetic adaptability (Pandolfo et al. 2024). Many of the genes involved in the degradation of anthropogenic contaminants originally evolved for distinct ecological functions, such as plant polymer degradation or secondary metabolism (Copley 2009). Horizontal gene transfer (HGT) and genome rearrangements have played a pivotal role in repurposing these genes for pollutant degradation, facilitating their rapid incorporation into catabolic networks and allowing microorganisms to use xenobiotics as carbon, nitrogen, or energy sources (Fenner et al. 2013) (Figure 1). These evolutionary mechanisms enable microbial communities to adapt efficiently to selective pressures imposed by environmental contaminants (Noda‐Garcia et al. 2018).

The assembly of XGCs exemplifies a contemporary manifestation of metabolite–enzyme coevolution, as proposed for the evolution of natural metabolic networks (Noda‐Garcia et al. 2018). In this context, pollutant molecules serve as novel selective pressures that drive the recruitment of promiscuous enzymes and promote the modular recombination of catabolic genes into coherent metabolic pathways (Figure 1). This dynamic highlights bioremediation not only as an ecological process but as an ongoing evolutionary process (an extension of metabolic network evolution), actively shaped by anthropogenic selection. These insights enhance our understanding of microbial adaptation mechanisms and their crucial role in improving bioremediation strategies in contaminated ecosystems.

Current‐omics approaches and metabolic engineering tools are now enabling the optimisation of these clusters and the design of specialised microbial consortia or ‘super‐degraders’ with enhanced efficiency for priority pollutants, offering a sustainable and cost‐effective alternative to conventional physicochemical remediation strategies (Bala et al. 2022; Narayanan et al. 2023; Sharma et al. 2022; Shah et al. 2024).

In this perspective article, we explore the evolutionary and ecological implications of these processes, emphasising their role in microbial cooperation, adaptation, and ecosystem resilience. We highlight the acquisition and horizontal transfer of catabolic pathways for pollutants and pharmaceuticals, discussing how understanding these mechanisms can inform strategies for optimising bioremediation, such as the engineering of microbial consortia and the targeted application of metabolic hubs.

## 2 Horizontal Gene Transfer and Adaptive Potential

HGT facilitates the exchange of genetic material between organisms independent of traditional vertical inheritance, acting as a powerful driver of microbial evolution. Through this process, microbes frequently exchange genes associated with antibiotic resistance (San Millán 2018), symbiosis (Wardell et al. 2022), and specialised metabolism (Saati‐Santamaría 2023), as well as those involved in the degradation of anthropogenic compounds (Stolz 2014). Consequently, HGT plays a central role in the adaptation of microbial communities to life in environments enriched with xenobiotics derived from human activity. In bacteria, HGT occurs primarily through conjugation, transformation, and transduction, and is mediated by a wide variety of MGEs, including plasmids, transposons, and bacteriophages (Soucy et al. 2015; Arnold et al. 2022; Penadés et al. 2025).

HGT is generally more efficient among closely related taxa, but the modular nature of MGEs also enables occasional transfers between distant lineages, creating opportunities for rapid evolutionary innovation (Caro‐Quintero and Konstantinidis 2015; Brito 2021; Haimlich et al. 2024). The long‐term persistence of a transferred gene depends on its functional integration into the host genome or its stable maintenance as a plasmid or integrative element, which ensures inheritance and incorporation into population‐level evolution (Baltrus 2013; Darmon and Leach 2014; Abe et al. 2020). Yet, whether such transfers persist or vanish depends not only on the architecture of the recipient genome but also on the balance of evolutionary forces acting upon them, such that HGT events providing a selective benefit are more likely to be retained, while others may be lost or maintained transiently (Berg and Kurland 2002; Wiedenbeck and Cohan 2011; Saati‐Santamaría et al. 2026).

Horizontally acquired elements can impose fitness costs that selection must compensate for over time (Baltrus 2013; Nguyen et al. 2022; Vos et al. 2024). Population genetics theory predicts that factors such as effective population size, strength of selection, and genetic drift critically determine the fate of transferred genes: in large populations, even mildly beneficial traits may be retained, while in small populations stochastic loss predominates (Charlesworth 2009; Arnold et al. 2022; Nguyen et al. 2022). Deciphering the complex evolutionary forces that govern HGT outcomes remains a major challenge (Andreani et al. 2017; Haudiquet et al. 2022; Galtier 2024), although ecological frameworks provide additional insights, suggesting that gene transfers are more likely to succeed when they confer traits that facilitate survival across ecological boundaries (Wiedenbeck and Cohan 2011; Zhaxybayeva and Nesbø 2025). Thus, HGT should be viewed not as a mere genetic accident but as an integral process within the eco‐evolutionary dynamics of microbial populations, driving diversification, facilitating ecological innovation, and balancing the adaptive potential and evolutionary burden.

The organisation of catabolic genes into operons plays a key role in facilitating HGT. The *selfish operon* model proposes that clustering functionally related genes enables entire pathways to be transferred as single units across different taxa (Lawrence and Roth 1996; Lawrence 1997, 1999). However, operon formation may also arise from regulatory or functional constraints independent of HGT, indicating that multiple selective pressures contribute to gene clustering in bacterial genomes (Pál and Hurst 2004; Price et al. 2005). Additionally, larger genetic elements such as plasmids often carry complete biosynthetic or catabolic pathways, allowing entire functional modules to transfer between distantly related bacteria, promoting rapid adaptation to new environmental pollutants (Stolz 2014; Aulestia et al. 2022; Saati‐Santamaría et al. 2025). Beyond simply moving genes, these HGT events may confer a selective advantage at the community level by increasing the number of microbial ‘workers’ capable of degrading challenging substrates. This perspective integrates the classical *selfish operon* concept with the broader evolutionary context of gene clustering and MGEs, emphasising that HGT‐mediated dissemination of functional traits operates at multiple genomic scales, from single operons to entire plasmids or Biosynthetic Gene Clusters.

Interestingly, not all HGT vectors are subject to the same selective constraints. For instance, while plasmids and transposons depend on host survival for their maintenance, bacteriophages often propagate through lytic cycles that destroy their hosts. Consequently, the fate of phage‐encoded genes is influenced not only by their contribution to viral replication but also by selective processes acting at host and community levels (Chen, Zhao, et al. 2024; Zhu et al. 2024; Zhou et al. 2025). This multi‐level selection underscores how ecological context can modulate the evolutionary outcomes of horizontal transfer.

In polluted environments, these same principles acquire an ecological dimension: the gene, operon, or mobile element itself can act as the unit of selection, persisting through mobility and ecological advantage even when the host cell is transient (Douglas and Shapiro 2021; Haudiquet et al. 2022). Operons encoding xenobiotic degradation pathways provide striking examples of this modularity in action (Mishra et al. 2001; Bhattacharyya et al. 2023; Saati‐Santamaría et al. 2025).

HGT is particularly relevant in environments impacted by human activity, where microorganisms face novel chemical stresses. Xenobiotics such as hydrocarbons, pesticides, plastics, and heavy metals create selective pressures that favour the acquisition and retention of degradation pathways (Top and Springael 2003; Nojiri et al. 2004; Springael and Top 2004; Goff et al. 2024). Wastewater treatment plants, hospital environments, and industrially impacted soils act as hotspots for genetic exchange (Kunhikannan et al. 2021; Hutinel et al. 2021). The well‐known situation with the spread of antibiotic resistance genes (ARGs) in environments with high antibiotic exposure is instructive. Just as ARGs are fixed globally in response to selective pressure (Wistrand‐Yuen et al. 2018; Baquero et al. 2021; Santos‐Lopez et al. 2021), many catabolic genes for pollutant degradation are found on plasmids and transposons, suggesting a high potential for gene mobility under increasing pollutant loads (Stolz 2014; Aulestia et al. 2022). Indeed, the selfish operon concept discussed above can similarly help explain the persistence and spread of both ARGs and xenobiotic degradation genes. In any case, while ARG transfer has been widely studied (Munk et al. 2022; Lund et al. 2025; Zhu et al. 2025), the global dynamics of HGT in response to emerging contaminants remain poorly understood. Understanding these processes is crucial for predicting microbial adaptation to anthropogenic pollutants.

Importantly, resistance to a xenobiotic is not necessarily equivalent to its catabolism: while resistance can allow survival in the presence of the compound, enzymatic degradation actively transforms or removes the molecule, often conferring an adaptive benefit to both individual cells and the broader community (Kehila et al. 2023, 2025). Xenobiotics often represent novel sources of carbon, nitrogen, or energy, creating opportunities for microbial growth and ecosystem‐level nutrient cycling. Beyond survival, these compounds can serve as nutrients or new sources of energy, opening ecological opportunities for microbes capable of transforming them (Noda‐Garcia et al. 2018). Recognising this dual role (protection and resource acquisition) helps explain why HGT‐mediated catabolic traits can rapidly spread in polluted environments.

In this context, the emergence and spread of *degradative plasmids* provide a compelling example of how conjugation drives functional adaptation in polluted environments. *Degradative plasmids* represent a particularly important class of MGEs in the context of bioremediation. These plasmids carry genes that confer the ability to metabolise and mineralise recalcitrant or xenobiotic organic compounds that are rarely encountered in uncontaminated natural environments. Classic examples include the TOL, CAM, and NAH plasmids, which encode pathways for the degradation of toluene, camphor, and naphthalene, respectively (Ikuma and Gunsch 2013; Menn et al. 1993; Rheinwald et al. 1973). Because they often contain complex operons organised into regulatory and catabolic modules, *degradative plasmids* can be readily mobilised between phylogenetically distant taxa through conjugation, rapidly spreading metabolic innovations across communities. Their maintenance is usually favoured in contaminated environments, where selective pressure by pollutants offsets the metabolic cost of carrying large plasmids. Over evolutionary timescales, genes from degradative plasmids can become integrated into the chromosome through homologous recombination, illustrating how episodic horizontal transfers contribute to the long‐term genomic adaptation of microbial lineages to anthropogenic compounds (Meulien and Broda 1982).

Beyond plasmids, bacteriophages can also contribute to the dissemination of metabolic traits under xenobiotic stress. Some aquatic phages encode auxiliary metabolic genes (AMGs) related to hydrocarbon degradation, such as virus‐encoded hydrocarbon degradation genes (*vHYDEG*), which transiently enhance host metabolism and survival during infection (Ru et al. 2023), thereby promoting phage replication and spread, providing a direct fitness advantage to the virus. Although phage‐mediated transfer is episodic, it adds a further eco‐evolutionary dimension to microbial adaptation in contaminated environments.

### 2.1 Metabolic HGT Hubs

**FIGURE 2:**
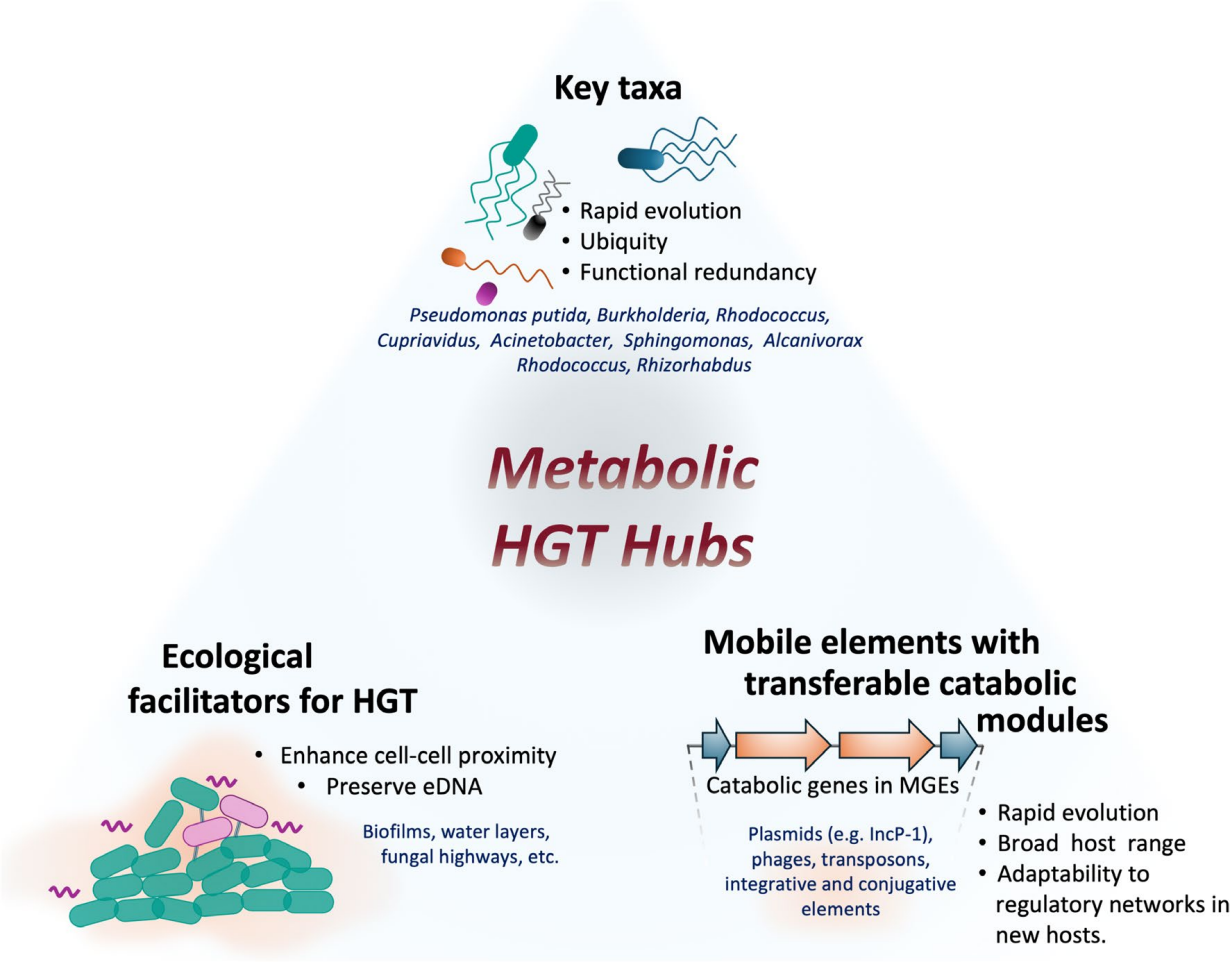
Key components of metabolic HGT Hubs enabling the emergence and spread of biodegradation traits. Metabolic horizontal gene transfer (HGT) Hubs arise from the interplay of three major components that together facilitate the rapid acquisition and dissemination of catabolic functions in microbial communities. *Mobile genetic elements carrying transferable catabolic modules*, *Key microbial taxa* acting as genetic donors, receivers, or community‐level biotransformation catalysts, and *Ecological facilitators for HGT*, such as high cell density, biofilm formation, and physicochemical microhabitats that promote DNA exchange and stabilise newly acquired traits. Together, these components create dynamic hotspots for the evolution of catabolic innovation and community‐level adaptation to anthropogenic compounds.

To conceptualise the role of HGT in shaping microbial communities, we introduce the term *Metabolic HGT Hubs* to refer to key microbial taxa or replicons that not only acquire degradation genes at high rates but also serve as central nodes promoting the assembly of novel degradation pathways and redistributing these genes within microbial consortia (Figure 2). These hubs, often associated with plasmids of broad host range (e.g., IncP‐1) or environmentally versatile bacteria (e.g., *Pseudomonas*, *Burkholderia*, *Rhodococcus*, *Cupriavidus*, *Acinetobacter*, and *Sphingomonas*), act as evolutionary nodes. Analogous to *keystone species* in ecological networks (Davic 2003; Wang et al. 2024), these hubs enhance the efficiency and adaptability of biodegradative communities by accelerating the spread of beneficial genetic traits for a more rapid functional innovation under selective pressure from xenobiotics.

The conceptual framework of *Metabolic HGT Hubs* is supported by a growing body of empirical evidence that reveals how specific microbial taxa and MGEs act as nodes of genetic redistribution in polluted environments. Among these, broad‐host‐range plasmids of the IncP‐1 group stand out as paradigmatic examples (Norberg et al. 2011; Klümper et al. 2015). Their ability to replicate across diverse Gram‐negative hosts and their unusually efficient conjugative machinery have made them central vectors of adaptive traits, ranging from antibiotic and metal resistance to the degradation of xenobiotic compounds (Norberg et al. 2011; Jechalke et al. 2013; Popowska and Krawczyk‐Balska 2013; Yakimov et al. 2016). Thus, IncP‐1 plasmids function as mobile hubs capable of disseminating multiple adaptive traits across phylogenetic boundaries.

Plasmids alone, however, do not account for the emergence of hubs; their ecological impact ultimately depends on the microbial hosts that maintain and propagate them. Several bacterial taxa repeatedly emerge as central players in this regard. For instance, members of the genus *Pseudomonas* represent paradigmatic examples of metabolic versatility and genomic plasticity among environmental bacteria. Their genomes are rich in MGEs, integrative conjugative elements, and accessory gene clusters that mediate rapid adaptation to fluctuating chemical landscapes (Silby et al. 2011; Saati‐Santamaría et al. 2022). Within this genus, *P. putida* can be pointed as an example of *Metabolic HGT Hub* and a model for understanding how catabolic innovation spreads across microbial communities. As de Lorenzo et al. (2024) highlights, the evolutionary trajectory of *P. putida* from a soil‐dwelling saprophyte to a synthetic‐biology chassis reflects its intrinsic ability to assimilate, reorganise, and redistribute genetic modules involved in the degradation of aromatic and xenobiotic compounds. *P. putida* carries plasmids of the IncP‐1 group, such as pWW0, pWW53, and pGRT1, which, together with integrative catabolic islands, provide a modular architecture that facilitates the acquisition and lateral dissemination of pathways for the breakdown of toluene, naphthalene, phenols, and chlorinated hydrocarbons (Segura et al. 2014). This connectivity places *P. putida* at the centre of metabolic gene exchange networks in polluted environments, where selective pressure from anthropogenic contaminants drives the retention and redistribution of biodegradation functions. Beyond its ecological role, the genetic malleability and regulatory robustness of *P. putida* make it an archetype of an HGT‐mediated metabolic hub, linking evolutionary adaptability with applied bioremediation potential.

Members of the *Burkholderia cepacia* complex (BCC) and related species within the genus *Burkholderia* also exemplify environmentally versatile bacteria that function as metabolic HGT hubs in contaminated environments. Their multipartite genomes, typically composed of two or three large chromosomes and one or more plasmids, encode an extensive array of catabolic, resistance, and stress‐response genes that enable survival in chemically diverse habitats (Pérez‐Pantoja et al. 2012; Mullins and Mahenthiralingam 2021). Numerous *Burkholderia* strains harbor conjugative plasmids and integrative conjugative elements (ICEs) carrying complete degradation pathways for chlorinated aromatics, phenols, herbicides, and polycyclic aromatic hydrocarbons. Their dual capacity for environmental persistence and genetic exchange positions *Burkholderia* as a pivotal evolutionary intermediary linking genomic plasticity with metabolic specialisation in bioremediation systems (O'Sullivan and Mahenthiralingam 2005).

Beyond *Burkholderia* and *P. putida* , additional taxa further exemplify the ecological and evolutionary dynamics that define *Metabolic HGT Hubs*. In particular, the *Novosphingobium*–*Sphingomonas* lineage is frequently found in sites contaminated with aromatic hydrocarbons (Leys et al. 2004; Saati‐Santamaría et al. 2025). In these environments, they host transferable plasmids encoding ring‐cleavage and other catabolic enzymes, and experimental work has demonstrated their ability to pass such plasmids across genus boundaries (Basta et al. 2004; Stolz 2014).

Also, in hydrocarbon‐contaminated communities, *Alcanivorax* and *Rhodococcus* strains are particularly abundant (Garrido‐Sanz et al. 2018; Kuyukina and Ivshina 2010), and studies have shown that the number of *alkB* copies—encoding the alkane monooxygenase essential for aerobic alkane degradation—increases in microbial populations exposed to petroleum, suggesting selective enrichment of alkane‐catabolic potential under chemical stress (Galitskaya et al. 2021). Many *Rhodococcus* strains encode multiple homologous *alkB* genes with distinct substrate specificities and regulatory controls (Whyte et al. 2002; Petrikov et al. 2025). The presence of these divergent yet functionally overlapping genes within single genomes points to repeated acquisition events via HGT, positioning *Rhodococcus* as a hub for alkane catabolic diversity, continuously reshaping its own genomic toolkit. Large‐scale genomic surveys further reveal that *alk* gene clusters are largely restricted to α‐ and γ‐proteobacteria and often flanked by insertion sequences, suggesting past transfers between taxa, although the mechanisms and ecological contexts of such transfers remain unclear (Wang et al. 2022).

*Rhizorhabdus* species have also been linked to the degradation of diverse environmental contaminants (Sánchez‐Arroyo et al. 2025). For instance, strains of *R. wittichii* carry plasmids containing the *ipf* gene clusters involved in ibuprofen biodegradation (Aulestia et al. 2022; Saati‐Santamaría et al. 2025). Remarkably, these *ipf* clusters show 100% nucleotide identity not only across different *Rhizorhabdus* strains but also in other taxonomically distant organisms such as *Sphingopyxis granuli* (Aulestia et al. 2022; Saati‐Santamaría et al. 2025). This conservation occurs despite differences in the surrounding plasmid context, suggesting that the *ipf* genes are highly mobilisable and may be exchanged between distinct replicons and species, maintaining their sequence integrity while the rest of the plasmid evolves independently. Indeed, these MGEs appear to be actively transferred among plasmids and host species within consortia engaged in ibuprofen metabolism (Saati‐Santamaría et al. 2025).

Overall, these cases exemplify the defining property of a Metabolic HGT Hub: the ability of metabolically versatile, ecologically dominant taxa to function not only as genetic reservoirs but also as distributors of catabolic pathways to other community members. Still, while in some cases dominant taxa may act as donors of catabolic genes to rarer community members, the reverse can also occur when rare carriers possess unique degradative capabilities. By transferring catabolic genes, hubs extend the adaptive capacity of the entire microbial community beyond the boundaries of individual lineages. However, transfer is constrained by factors such as phylogenetic distance, compatibility of regulatory and metabolic networks, and ecological interactions, with closely related or co‐occurring species being more likely recipients (Baltrus 2013; Popa and Dagan 2011). These biological and ecological filters shape the effectiveness of hubs in promoting community‐level adaptation under selective pressures from xenobiotic compounds, ultimately linking genomic plasticity with ecosystem function.

The operation of these hubs is not only a genetic but also an ecological phenomenon (Figure 2). Environmental factors can either hinder or amplify the efficiency of HGT, and therefore the hub dynamic itself (Acar Kirit et al. 2022). In unsaturated soils, for instance, the discontinuity of water films severely restricts cell‐to‐cell contact and limits conjugation. Yet filamentous fungi overcome this barrier by growing across air‐filled pores, generating continuous liquid films along their hyphae that serve as ‘fungal highways’ for bacterial dispersal (Kohlmeier et al. 2005). The resulting microhabitat, termed the *mycosphere*, is enriched by fungal exudates and functions as a physical scaffold where bacterial cells accumulate, interact, and exchange genetic material at elevated frequencies (Frey‐Klett et al. 2011; Zhang et al. 2014; Pratama and Van Elsas 2018). These fungal‐bacterial interfaces provide the ecological architecture required for hubs to achieve their full potential in situ, effectively transforming structural heterogeneity into hotspots of adaptive innovation.

A similar principle applies to bacterial biofilms, which represent densely packed, matrix‐embedded communities where physical proximity, nutrient gradients, and signalling molecules collectively enhance the frequency of gene transfer events. Within biofilms, conjugative plasmids, integrative elements, and even membrane vesicles can circulate more efficiently than in planktonic populations (Abe et al. 2020; Hausner and Wuertz 1999; Madsen et al. 2012; Molin and Tolker‐Nielsen 2003; Soler et al. 2015). Moreover, the biofilm matrix itself, composed of extracellular DNA, polysaccharides, and proteins, provides both a scaffold and a genetic reservoir that sustains horizontal exchange over extended periods (Flemming and Wingender 2010). In this sense, biofilms act as self‐organised ecological incubators of HGT, transforming surface‐associated microbial assemblages into persistent hotspots of genetic innovation and metabolic diversification.

Taken together, these lines of evidence define *Metabolic HGT Hubs* not as single organisms or plasmids but as dynamic systems integrating three dimensions: ecologically dominant microbial taxa, broad‐host‐range MGEs that carry transferable catabolic functions, and ecological facilitators that enhance opportunities for contact and exchange. The convergence of these dimensions likely explains why the distribution of catabolic genes so often mirrors habitat characteristics rather than evolutionary lineage.

### 2.2 Metabolic HGT Hubs in Applied Bioremediation Strategies

Beyond the natural spread of catabolic functions, microbial communities can also be deliberately engineered to enhance biodegradation. Introducing MEGs carrying complete degradation pathways can boost the adaptative potential of natural microbes, tailoring microbiomes to polluted environments (Gao et al. 2015; Yip et al. 2024; Marquiegui‐Alvaro et al. 2025). The implications of *Metabolic HGT Hubs* for applied bioremediation are profound. Conventional bioaugmentation strategies often focus on introducing degrader strains, yet these inoculants frequently fail due to poor competitiveness against indigenous microbiota (Garbisu and Alkorta 2003).

A hub‐based perspective shifts the emphasis toward *genetic bioaugmentation*. Rather than introducing a strain expected to dominate the system, this strategy focuses on deploying proven genetic donors equipped with broad‐host‐range plasmids—such as those of the IncP‐1 group—capable of disseminating catabolic modules into indigenous populations (Pepper et al. 2002). In this framework, the donor acts as a seed within a *Metabolic HGT Hub*, enabling adaptive functions to spread across phylogenetically diverse hosts that are already ecologically competitive in situ. This process can be further enhanced by providing physical scaffolds or support materials that promote biofilm formation, thereby creating the high‐density hotspots required for efficient gene transfer. By leveraging the intrinsic tendency of contaminated ecosystems to reorganise their genomic architecture through gene acquisition and subsequent stabilisation, genetic bioaugmentation may provide an evolutionary shortcut that accelerates community‐level adaptation to anthropogenic pollutants.

At the same time, the dual‐use nature of MGEs necessitates caution. The same plasmids that distribute catabolic genes frequently co‐transfer resistance to antibiotics and heavy metals, raising the possibility that large‐scale interventions could inadvertently exacerbate the spread of antimicrobial resistance (Heuer and Smalla 2012). The challenge for future applications will be to harness the adaptive potential of hubs while mitigating these risks, ensuring that bioremediation strategies deliver ecological and public health benefits in tandem.

All in all, HGT represents an evolutionary shortcut: it can rapidly assemble functional systems that have already evolved and are immediately operative under diverse environmental conditions. Although risky—since the introduction of foreign elements may disrupt existing regulatory networks—the evolution of entirely new beneficial genes is both slow and rare, making sharing often more efficient than reinventing. Beyond simply moving coding sequences, HGT can reshape gene expression, generating higher, lower, or constitutive activity, or even placing genes under new regulatory controls (Darmon and Leach 2014).

## 3 Genome Rearrangements and Functional Optimisation

Beyond gene acquisition by HGT, genome rearrangements such as duplications, insertions, deletions, and inversions, as well as single nucleotide polymorphisms (SNPs) contribute to the gaining of new metabolic pathways or to their fine‐tuning. Collectively, these modifications can contribute to the biodegradation capacity of microbial communities by increasing enzyme abundance, modulating transcriptional regulation, improving metabolic flexibility and restructuring operons and catabolic pathways. Structural genome changes can also promote specialisation within microbial communities, leading to the emergence of cooperative interactions where different species optimise complementary functions (Bedhomme et al. 2019; Martín‐González et al. 2024; Ren and Manefield 2025; Saati‐Santamaría et al. 2025).

From an evolutionary perspective, genome rearrangements provide the structural framework that enables transitions between generalist and specialist enzyme functions. Promiscuous activities of pre‐existing enzymes supply the raw material for new catalytic functions (Noda‐García et al., 2018). When such activities confer a selective advantage—such as activity toward a novel xenobiotic substrate—they may be refined through gene duplication. Typically, the original gene retains its ancestral function, while the duplicated copy accumulates mutations that lead to specialisation, a process known as subfunctionalisation (Sélem‐Mojica et al. 2019).

*Rhodococcus jostii* RHA1 serves as an important reference model for dissecting how bacteria adapt to contaminated environments through dynamic genome restructuring that expands and refines their catabolic capabilities for complex aromatic pollutants. Its ~9.7 Mb genome is comprised by a linear chromosome and three large linear plasmids (Larkin et al. 2005, 2006; McLeod et al. 2006). Such expanded genomic architecture provides a versatile platform for acquisition, rearrangement, and retention of biodegradation functions. Notably, RHA1 harbours duplicated catabolic islands that contains the *pad*/*pat*/*tpa* gene clusters involved in phthalate/terephthalate metabolism. These clusters are present on both linear plasmids pRHL1 and pRHL2. Additionally, numerous insertion sequences on plasmids create recombination hotspots, enabling operon shuffling and functional innovation. Their duplicated organisation and flanking mobile elements indicate horizontal gene acquisition followed by internal duplication and rearrangement (McLeod et al. 2006; Patrauchan et al. 2005). RHA1 also encodes over 200 monooxygenases and cytochrome P450 enzymes, many plasmid‐borne, reflecting substantial gene family expansion and duplication. These provide metabolic flexibility enabling degradation of structurally diverse xenobiotics, including polynuclear aromatics and substituted aromatics (McLeod et al. 2006). Proteomic and transcriptomic data shown biochemical redundancy and alternative flux pathways under conditions that favour particular downstream enzymes. These findings help explain how duplicated enzyme families and multiple oxygenases in RHA1 contribute to metabolic plasticity, rather than simply genomic redundancy (Spencer et al. 2020).

The biodegradation of polycyclic aromatic hydrocarbons (PAHs) is another context in which bacteria gain and expand their capabilities through the acquisition, rearrangement, and mobilisation of catabolic operons, largely mediated by MGEs. A well‐studied case is the IncP‐9 plasmid NAH7 found in *Pseudomonas putida* G7, which carries the *nah* operons for naphthalene degradation. These genes reside within the transposon Tn4655, which encodes a tyrosine recombinase that enables excision and integration of the entire catabolic gene cluster into different replicons (Sota et al. 2006). This modular transfer mechanism accelerates the spread of degradation functions across microbial communities, underscoring genetic mobility as a fundamental evolutionary strategy in polluted environments.

Integrative and Conjugative Elements (ICEs) represent another major vehicle of genomic restructuring. In *Pseudomonas stutzeri* KF716, an ICE of the ICEclc subfamily carries the *bph* (biphenyl) and *sal* (salicylic acid) operons associated with aromatic degradation (Hirose et al. 2021). These ICEs can excise from the chromosome, conjugate, and integrate into recipient genomes, thereby stabilising and spreading catabolic functions across bacterial populations. Although focused on biphenyl/PCBs, similar ICEs mediate PAH degradation in related systems, enhancing bacterial adaptation to polluted environments. These elements often harbour gene clusters that encode complete pathways for aromatic degradation, including dioxygenases and monooxygenases, which are key for the breakdown of complex hydrocarbons.

Microbial adaptation to anthropogenic contaminants often relies on regulatory innovations that enable latent or promiscuous enzymes to interact with novel substrates. Spontaneous mutations, such as SNPs in transcriptional regulators, deletions that disrupt repressor networks, and genomic rearrangements creating new promoter architectures, can enhance the expression of catabolic genes, ultimately improving biodegradation efficiency (Deng et al. 2019; Gao et al. 2016; Haq et al. 2024). For example, during adaptive laboratory evolution, *Rhodopseudomonas palustris* acquired the ability to metabolise 3‐chlorobenzoate through the convergence of two spontaneous mutations: (i) a large deletion removing the transcriptional repressor BadM, which derepressed aromatic CoA‐ligase catabolic pathways, and (ii) a SNP in AliA that enhances catalytic efficiency toward the chlorinated substrate (Haq et al. 2024). The evolved strain expresses the degradation pathway constitutively, bypassing the requirement for a native inducer and enabling efficient turnover of a previously non‐metabolisable compound. In parallel with these experimental observations, metagenomic analyses of contaminated environments consistently show that catabolic genes are frequently associated with mobile genetic elements, highlighting the central role of horizontal gene mobilisation in shaping microbial degradation capacities in natural settings (Ghosal et al. 2016; Liang et al. 2019).

Another clarifying example comes from the regulatory adaptation of environmental microbial consortia for the biodegradation of ibuprofen. It has been shown that adaptation to IBU involves not only the acquisition of catabolic genes but also extensive regulatory remodelling within microbial communities (Saati‐Santamaría et al. 2025). Key regulatory mutations must have facilitated the induction and upregulation of previously latent pathways, enabling efficient mineralisation of IBU at environmentally relevant concentrations. Moreover, cross‐species regulatory interactions within the consortia fine‐tuned gene expression networks, enhancing metabolic cooperation and substrate turnover. These results highlight that beyond genetic acquisition, regulatory innovation is critical for the functional integration and optimisation of xenobiotic degradation pathways in complex environmental microbiomes (Saati‐Santamaría et al. 2025).

Collectively, these studies suggest that genomic rearrangements, at both the bacterial strain and community level, are central to microbial adaptation to xenobiotics and a key evolutionary mechanism that enables bacteria to acquire, refine, and coordinate catabolic functions in contaminated environments.

## 4 Microbial Cooperation and Ecosystem Functioning

Microbial consortia are pivotal in the biodegradation of complex compounds, where cooperation among species enhances metabolic efficiency and ecosystem resilience. Recent studies provide strong evidence supporting cooperative interactions within consortia as key drivers of effective biodegradation (Sanchez et al. 2023; Li et al. 2022; Pascual‐García et al. 2020; Lü et al. 2024).

One central theory explaining this phenomenon is the concept of *metabolic division of labour*, where different microbial species specialise in distinct steps of a degradation pathway (Tsoi et al. 2018) (Figure 1). This syntrophic cooperation allows consortia to break down complex pollutants more efficiently than individual strains acting alone, as one species might convert a contaminant into intermediate metabolites, which are then further degraded by other members, forming a degradation cascade that maximises resource utilisation and energy yield.

A key mechanism supporting this is HGT‐mediated metabolic complementation, whereby plasmids and other MGEs transfer catabolic modules between species. While MGEs often carry complete metabolic operons, their distribution across a consortium can lead to metabolic specialisation through differential gene loss and streamlining, processes that minimise the fitness cost of maintaining long enzymatic cascades in a single host, promoting stable cross‐species cooperation by partitioning the degradation pathway into distinct cellular nodes. This genetic plasticity not only expands functional repertoires but also supports rapid evolution and resilience under changing environmental conditions (Tokuda and Shintani 2024). This cooperative architecture is reinforced by selective pressures imposed by pollutants, which favour microbial groups capable of dynamic gene exchange and resource sharing.

At the community level, ecological theories such as the keystone species and functional redundancy concepts emphasise the importance of microbial community structure. Keystone species, often highly connected taxa within microbial networks, maintain functional interactions that stabilise ecosystem processes (Cavaliere et al. 2017; Niehaus et al. 2019; Jiménez‐Volkerink et al. 2024). Functional redundancy ensures ecosystem resilience by providing backup metabolic functions when some species are lost or environmental stresses occur (Cavaliere et al. 2017; Niehaus et al. 2019).

Plasmids carry gene clusters encoding enzymes responsible for the degradation of various organic pollutants, including PAHs, pesticides, and pharmaceuticals (Bhatt et al. 2021; Huang et al. 2025; Mohapatra and Phale 2021). These plasmid‐borne gene clusters often include catabolic genes for enzymatic breakdown, transport genes for pollutant uptake, and regulatory genes for pathway expression, enabling microbes to adapt and cooperate effectively in contaminant degradation processes.

In consortia degrading PAHs, genera such as *Sphingomonas* and *Pseudomonas* have been shown to harbour plasmids encoding ring‐hydroxylating dioxygenases and other critical catabolic enzymes (Jiménez‐Volkerink et al. 2024; Ma et al. 2006; He et al. 2018; Ghosal et al. 2016; Mohapatra and Phale 2021). These plasmids enable the host bacteria to metabolise complex hydrocarbons and pass these capabilities to other species, supporting syntrophic degradation networks. The dynamic movement of plasmids allows different species to acquire complementary metabolic functions essential for complete degradation pathways, facilitating cooperation where individual isolates cannot perform all degradation steps independently (Tokuda and Shintani 2024).

Adaptation within microbial consortia also involves shifts in species composition or relative abundance, often driven by the selective pressures exerted by pollutants and facilitated by the exchange of genetic material. Genome rearrangements at the community scale underpin metabolic complementation, where species dynamically adjust to fill metabolic niches created by cooperative degradation pathways (Chen, Wang, et al. 2024).

Beyond plasmid transfer, intrinsic genome rearrangements such as gene duplications, insertions/deletions, and transpositions contribute to the diversification of metabolic functions within consortia. These structural genomic changes facilitate the evolution of novel catabolic pathways and the adaptive fine‐tuning of enzymatic functions in response to environmental fluctuations. For example, rapid genome rearrangements in microbial consortia degrading pharmaceuticals such as ibuprofen have been shown to have taken place, where duplications and MGEs mediate functional diversification and enhanced pollutant breakdown capacity (Saati‐Santamaría et al. 2025).

Altogether, plasmid‐mediated HGT and genome restructuring contribute to metabolic complementarity and ecological resilience. This genomic and ecological plasticity represents a fundamental evolutionary mechanism sustaining the efficiency and persistence of biodegradation processes in both natural and engineered environments.

## 5 Selective Pressures and Evolutionary Trajectories in Biorremediation

Pollutant exposure creates strong selective pressures that drive microbial evolution (Copley 2009). Microbial populations involved in bioremediation evolve under intense and often multifactorial selective pressures that shape their genomic architecture and ecological behaviour. Contaminated environments act as evolutionary laboratories where chemical stressors, nutrient imbalances, and microbial interactions accelerate genome rearrangements and HGT. Importantly, these chemical stressors operate within complex ecological matrices where pollutants coexist with a wide range of natural substrates, including carbohydrates, organic acids, and recalcitrant polymers such as cellulose and lignin. In this context, pollutants do not act in isolation but as additional components within multi‐resource environments, where native carbon sources and nutrient availability continue to play a major role in shaping community structure and metabolic activity. Thus, the evolutionary trajectories of microbial populations reflect not only adaptation to contaminants but also their integration into pre‐existing ecological and metabolic networks. In contaminated environments, microbes with enhanced genetic plasticity are more likely to survive and proliferate (Tokuda and Shintani 2024). The frequent exchange of degradation genes within these populations can lead to the emergence of novel metabolic pathways (Noda‐Garcia et al. 2018). While these processes enhance biodegradation, they can also disrupt native microbial communities by altering ecological balances. The uncontrolled spread of degradation genes may lead to unintended metabolic interactions, potentially destabilising microbial networks or interfering with pre‐existing ecological functions (Pandey et al. 2009). Thus, this rapid evolution highlights the need to consider ecological and evolutionary dynamics when designing bioremediation strategies.

These patterns are consistent with the *environmental filtering hypothesis*, which posits that environmental conditions act as filters selecting for organisms with traits that confer tolerance or efficiency under specific stress regimes (Weiher and Keddy 1995). Although this concept has been mainly applied in plant ecology, some studies have tested this hypothesis in bacterial communities (Freedman and Zak 2015; Yan et al. 2019; Zhou et al. 2021). In polluted ecosystems, chemical stress and nutrient limitation operate as strong filters that narrow community composition to taxa with robust stress‐response systems, efficient efflux mechanisms, or expanded catabolic repertoires (Yan et al. 2019). This filtering effect not only determines which lineages persist but also shapes the direction of evolutionary change, favoring the retention and horizontal dissemination of adaptive genetic modules.

In the medium to long term, microbial lineages under chronic pollutant exposure may follow two distinct trajectories. Some evolve toward *genomic expansion*, accumulating redundant mobile elements that confer flexibility and rapid adaptability to fluctuating stressors (Props et al. 2019). This expansion phase enhances exploratory capacity, enabling rapid metabolic innovation and facilitating the assembly of novel degradation pathways through horizontal gene transfer. In highly dynamic or chemically heterogeneous environments, such expanded genomes may confer a decisive adaptive advantage by allowing flexible responses to fluctuating stressors. Also, some lineages may undergo *genome streamlining*, integrating key catabolic pathways into their chromosomes while discarding accessory genes to reduce metabolic burden (Giovannoni et al. 2014). Both strategies represent adaptive trade‐offs between innovation and stability, reflecting the evolutionary tension that defines microbial life in anthropogenic habitats.

However, evolution is determined by both biotic and abiotic factors simultaneously. At the community level, selective pressures are shaped by microbial cooperation and competition. Bioremediation processes frequently occur in structured habitats—biofilms, soil aggregates, rhizospheric niches—where cell density and proximity facilitate gene exchange (Brito 2021). Under these conditions, genes conferring tolerance to xenobiotics, oxidative stress, or enhanced biofilm formation can spread rapidly, transforming transient interactions into stable metabolic partnerships. These ecological filters channel evolutionary trajectories toward *division of labour* and metabolic complementarity within consortia (Lenski 2017).

A complementary adaptive pattern emerging from these dynamics is *functional redundancy*, a hallmark of microbial systems exposed to chronic selective stress. The acquisition of redundant metabolic functions through HGT and genome rearrangements can have profound evolutionary consequences. *Functional redundancy*, where multiple species or genetic elements perform overlapping biochemical roles (Louca et al. 2018), can provide ecosystem resilience by buffering against environmental fluctuations (Ramond et al. 2025). This redundancy can arise from the horizontal acquisition of catabolic genes or through genome rearrangements that lead to gene duplication and neofunctionalisation. *Functional redundancy* is not only observed at the community level but also within individual genomes. The presence of multiple copies of genes encoding similar enzymatic functions within a single genome can provide metabolic flexibility, allowing bacteria to fine‐tune their response to fluctuating environmental conditions. Gene duplications followed by subfunctionalisation or neofunctionalisation may contribute to the diversification of degradation pathways, ensuring adaptability to different pollutant structures and concentrations. For instance, the bacterium *Rhodococcus* sp. RHA1, a well‐known degrader of xenobiotics, exemplifies this intragenomic redundancy. Its genome contains multiple gene copies for key enzymes, such as ring‐cleavage dioxygenases, which are crucial for degrading aromatic hydrocarbons. The presence of several genes with similar functions allows the bacterium to degrade a wider variety of PAHs and adapt to different concentrations, showcasing how gene duplication and subsequent specialisation enhance metabolic versatility (Larkin et al. 2005).

From an evolutionary perspective, *functional redundancy* allows for the maintenance of genetic diversity, increasing the likelihood of adaptive innovations. In polluted environments, redundancy in biodegradation pathways may help microbial consortia retain their degradative capacity even when environmental conditions change or specific taxa are lost. For example, microbial communities involved in hydrocarbon and aromatic compound degradation often exhibit overlapping enzyme functionalities across different taxa, such as multiple alkane monooxygenases (AlkB) and catechol dioxygenases (CatA) encoded by distinct species within the same consortium (Van Beilen et al. 2003; Louca et al. 2018). This redundancy enables metabolic plasticity, allowing communities to shift their catabolic strategies in response to variations in pollutant concentration or composition. Similarly, in wastewater treatment systems, multiple bacterial species harbouring genes for the degradation of pharmaceuticals and surfactants contribute to process stability by distributing the metabolic load and preventing functional collapse during environmental perturbations (Chen et al. 2020; Chen, Wang, et al. 2024). Such redundancy represents a form of ecological insurance, maintaining the continuity of biodegradation processes while providing a genetic reservoir from which novel catabolic variants may emerge through recombination and HGT. Importantly, this redundancy may also create the ecological and evolutionary conditions that favour metabolic interdependence and adaptive gene loss within consortia.

The persistence of these complex degradative networks can be further interpreted through the lens of the *Black Queen Hypothesis* (BQH), originally proposed to explain adaptive gene loss and metabolic interdependence in microbial communities (Morris et al. 2012). Within the context of bioremediation, we propose that the degradation of complex pollutants frequently operates as a ‘leaky’ public good. In what can be conceptualised as a metabolic HGT hub, not every individual lineage needs to encode the complete enzymatic toolkit required for full mineralisation. Instead, through metabolic division of labour, different species specialise in distinct steps of the degradation cascade, collectively sustaining the overall catabolic process.

In this framework, *functional redundancy* may lower the selective cost of gene loss within consortia, thereby facilitating BQH‐like dynamics. When multiple community members encode overlapping catabolic functions, the persistence of those functions no longer depends on every lineage maintaining a complete enzymatic repertoire. This buffering effect creates ecological conditions under which adaptive gene loss becomes selectively viable, particularly for energetically costly or regulatory complex pathways. In other words, as long as certain members of the consortium maintain energetically costly but essential ‘leaky’ functions (such as the initial oxidation or ring‐cleavage of a recalcitrant compound), other community members can undergo genome streamlining, losing these same genes to reduce their metabolic burden while still benefiting from shared intermediates. This *division of labour* generates a cooperative landscape in which *gene loss* is not necessarily detrimental but represents an adaptive strategy enabled by community context. HGT may further stabilise this system by allowing the redistribution or re‐acquisition of key catabolic modules when environmental conditions shift. Thus, HGT‐mediated complementation and selective gene loss may coexist as intertwined evolutionary forces in contaminated environments. Rather than every lineage maximising its individual catabolic repertoire, the community as a whole can maintain high degradative efficiency at a reduced per‐genome cost. This dynamic enhances ecosystem resilience while simultaneously increasing interdependence among taxa, reinforcing the view that successful bioremediation emerges from evolutionary and ecological integration rather than from the capabilities of isolated ‘super‐degrader’ strains. However, these cooperative dynamics do not occur in isolation but are embedded within broader ecological constraints that ultimately determine which lineages persist.

Bacterial evolution in contaminated environments is strongly shaped by ecological constraints rather than by genetic potential alone. In these environments, competition for limited resources and niche saturation by resident microbiota often restrict the establishment of incoming degraders, even when they possess suitable catabolic pathways. Čaušević et al. (2024) demonstrated that inoculated degraders such as *Pseudomonas veronii* experience ‘competitive loss by facilitation’, whereby their metabolic activity releases intermediates that are subsequently exploited by resident taxa, thereby reducing the inoculant's competitive advantage within an already resource‐saturated resident community. This dynamic underscores that contaminated soils act as complex scenarios of metabolic redistribution, where pollutants can simultaneously function as selective agents and shared resources. Thus, adaptive success depends not only on the acquisition or transfer of degradative genes but also on the ecological architecture that determines access to nutrients, cooperation, and competition within the microbial network.

Evaluating these risks is crucial for developing sustainable bioremediation strategies that maximise efficiency while minimising unintended consequences.

## 6 Future Directions and Concluding Remarks

The future of bioremediation research will depend on a deeper understanding of the ecological and evolutionary dynamics that govern bacterial adaptation to pollutants. Contaminated environments are not only sites of degradation but also evolutionary landscapes where selective pressures, gene flow, and metabolic cooperation continuously reshape bacterial communities. To predict and eventually guide these processes will be essential to integrate ecological theory with genomic and metabolic data, identifying the environmental variables and community architectures that favour the emergence and maintenance of catabolic traits.

Within this ecological framework, a major research goal is to identify and harness naturally evolved metabolic systems that already possess the enzymatic and regulatory capacities required for pollutant degradation. Many of the most efficient biodegradative pathways—such as those for hydrocarbons, aromatics, or pharmaceuticals—have originated in bacteria that inhabit chronically impacted ecosystems or environments rich in natural xenobiotic analogs (Mishra et al. 2001; Janssen et al. 2005). Mapping the ecological and genomic distribution of these catabolic systems will reveal microbial lineages, plasmids, and gene clusters with high potential for functional robustness and transferability. Linking this exploration with environmental genomics can guide the targeted enrichment or engineering of bacterial communities optimised for specific pollutants (Fabryová et al. 2018).

The convergence of Synthetic Biology and genetic engineering provides a critical pathway to overcome the limitations of natural bioremediation by enabling the design and optimisation of tailor‐made bacterial strains, or synthetic inoculants. This approach focuses on constructing stable and robust microbial chassis equipped with enhanced or novel catabolic functions (Lv et al. 2022). The main technical challenge lies in integrating complex catabolic modules—often derived from natural MGEs—into the host's genome or into designed, non‐transmissible plasmids, ensuring optimal expression and long‐term stability within the contaminated ecosystem. Key strategies include: (1) metabolic pathway optimisation through gene editing tools like CRISPR‐Cas to remove bottlenecks and increase degradation rates; (2) designing robust regulatory systems that only activate the degradative pathway in the presence of the pollutant, thereby minimising metabolic burden and promoting persistence; and (3) creating synthetic consortia where microbial strains are rationally programmed to cooperate, dividing complex degradation tasks or engaging in cross‐feeding metabolism (Grandel et al. 2021; Lv et al. 2022). However, the application of such synthetic inoculants in the environment requires a profound understanding of their genetic stability, balancing efficacy with the principles of biosafety and biocontainment to prevent the unintended horizontal transfer of engineered genes to native microbiota.

While these discoveries open new possibilities for environmental biotechnology, they also raise important biosafety and stability concerns. The same mechanisms that promote adaptation can facilitate the unintended dissemination of mobile elements carrying resistance or virulence genes. As bioremediation increasingly relies on engineered consortia, ensuring their ecological compatibility and genetic containment will be crucial. Future strategies should therefore combine evolutionary foresight with risk assessment, designing interventions that evolve within safe ecological boundaries.

A subsequent frontier lies in the identification and characterisation of evolutionary hotspots—ecological and genomic contexts where gene transfer and recombination occur at high frequencies. These sites are particularly relevant because many genetic innovations arise there, driving microbial adaptation and diversification. Despite remarkable advances, current metagenomic assemblies still struggle to accurately reconstruct MGEs and assign plasmids to their host taxa, limiting our capacity to trace gene flow and evaluate its ecological impact. Long‐read sequencing, single‐cell genomics, and proximity ligation techniques are beginning to overcome these barriers, enabling a more precise view of how catabolic functions spread within bacterial populations.

Beyond gene acquisition, adaptive gene loss and the emergence of metabolic streamlining will also shape bioremediation outcomes. The ability of bacteria to discard or repress costly pathways under low pollutant loads may determine the long‐term persistence of degradative traits in natural and engineered systems. Understanding this balance between innovation and reduction is essential for predicting community stability.

Finally, bioremediation faces the escalating challenge of emerging and recalcitrant pollutants—compounds such as microplastics, PFAS, and pharmaceuticals that have no evolutionary precedent. Their structural novelty demands new enzymatic solutions and cooperative metabolic networks that cannot be derived from single organisms. Combining evolutionary ecology studies with synthetic biology, and adaptive laboratory evolution may accelerate the development of novel catabolic routes while maintaining compatibility with natural ecosystems.

Taken together, these perspectives highlight that understanding bioremediation through the lens of metabolic network evolution reveals it as a contemporary extension of life's adaptive repertoire. For bacteria, pollutants do not represent merely environmental stressors but also novel ecological opportunities: sources of carbon, nitrogen, sulphur, and energy that can be exploited through metabolic innovation. HGT, genome rearrangements, SNPs, and ecological filtering collectively drive the continuous remodeling of bacterial metabolism under anthropogenic selection, transforming contaminants into selective landscapes that favor the emergence of new catabolic functions. In this way, bioremediation exemplifies not only the restoration of ecosystem function but also the expansion of bacterial metabolic potential in response to human‐induced chemical diversity.

Beyond gene acquisition, these processes underpin the emergence of metabolic cooperation, where distinct microbial species partition and exchange catabolic functions to collectively degrade complex pollutants. Such division of labour transforms communities into distributed metabolic networks, in which the success of bioremediation depends not only on the genetic potential of individual strains but also on their ecological interactions and exchange of mobile elements. In this sense, microbial consortia engaged in pollutant degradation embody the same evolutionary principles that shaped ancient metabolic networks: modularity, redundancy, and coevolution between enzymes and metabolites.

Viewing microbial communities as eco‐evolutionary systems rather than static tools reframes bioremediation as a process of ongoing adaptation. *Metabolic HGT hubs* and fluctuating selective pressures contribute to a self‐organising framework that continuously redistributes catabolic potential across taxa. Understanding and harnessing these dynamics will be essential for designing future bioremediation strategies that balance efficiency with biosafety, leveraging natural evolutionary mechanisms to sustain environmental recovery in a rapidly changing world.

## Author Contributions

**Inés Canosa:** writing – original draft, writing – review and editing. **Paula García‐Fraile:** writing – review and editing, conceptualization, writing – original draft. **Amando Flores:** writing – original draft, writing – review and editing. **Zaki Saati‐Santamaría:** conceptualization, writing – original draft, visualization, writing – review and editing.

## Funding

This work was supported by Escalera de Excelencia (CLU‐2025‐2‐04), Consejería de Educación de Castilla y León, FEDER Funds 2021–2027, Program EU Horizon Europe (HORIZON‐TMA‐MSCA‐PF‐EF) (101090267), MCIU/AEI (RYC2023‐045204‐I), MCIN/AEI (CEX2020‐001088‐M), Programa de Excelencia de la Junta de Andalucía (ProyExcel_00358), Plan Complementario de I+D+I, Plan de Recuperación, Transformación y Resiliencia (BIOD22_00033_20_PPCB. AGROREG), Programa Estatal Para la Investigación y el Desarrollo Experimental 2024–2027 (PID2024‐159973OB‐I00) and University Pablo de Olavide.

## Conflicts of Interest

The authors declare no conflicts of interest.

## Data Availability

Data sharing not applicable to this article as no datasets were generated or analysed during the current study.
